# Artificial Intelligence in Pediatric Inflammatory Bowel Disease: Applications in Diagnosis, Monitoring, and Therapeutic Decision-Making

**DOI:** 10.3390/children13020260

**Published:** 2026-02-13

**Authors:** Guilherme Dias Cabaço, Luís Rodrigues

**Affiliations:** 1 Lisbon School of Medicine, University of Lisbon, 1649-028 Lisbon, Portugal; gcabaco@edu.ulisboa.pt; 2 Gastroenterology Unit, Pediatrics Department, Hospital de Santa Maria, Unidade Local de Saúde Santa Maria, 1649-035 Lisbon, Portugal

**Keywords:** inflammatory bowel disease, Crohn’s disease, ulcerative colitis, artificial intelligence, machine learning, deep learning, pediatric

## Abstract

**Highlights:**

**What are the main findings?**

- Artificial intelligence applications in pediatric inflammatory bowel disease show promising performance, particularly in image-based and multimodal assessment.
- Pediatric-specific evidence remains limited, with many studies relying on adult-derived data.

**What are the implications of the main findings?**

- Artificial intelligence may support standardized, non-invasive, and longitudinal disease assessment in pediatric inflammatory bowel disease.
- Clinical translation requires pediatric validation, explainable models, and integration into care pathways.

**Abstract:**

**Background**: Pediatric inflammatory bowel disease (IBD) is characterized by a heterogeneous and often aggressive disease course, requiring complex multimodal assessment and long-term monitoring. Artificial intelligence (AI) has emerged as a promising tool to support clinical decision-making by enabling an objective analysis of large, multidimensional datasets. **Objectives**: This narrative review aims to critically synthesize current evidence on the application of AI across the diagnosis, monitoring, and treatment of pediatric IBD. **Methods**: A narrative literature review was conducted using the PubMed (MEDLINE) and Cochrane Library databases, including publications available up to December 2025. Pediatric-focused studies were prioritized. However, due to the limited availability of pediatric-specific AI research, a considerable proportion of the evidence reviewed derives from adult or mixed cohorts, which were included when methodological frameworks or clinically relevant endpoints were applicable to pediatric IBD. Eligible publications included narrative and systematic reviews, observational studies, and clinical trials focusing on AI applications in endoscopy, histology, imaging, disease monitoring, and therapeutic response prediction. **Results**: AI-based models, particularly those using machine learning and deep learning, demonstrated promising performance in the automated analysis of endoscopic, histological, and imaging data, reducing interobserver variability and improving workflow efficiency. Multimodal approaches integrating imaging, clinical, and biomarker data consistently outperformed unimodal models. Emerging applications in patient-centered monitoring, digital biomarkers, and telemedicine enabled continuous disease assessment and early detection of flares, with particular relevance in pediatric settings where repeated, non-invasive monitoring is essential. AI-driven models also showed promising accuracy in predicting therapeutic response, supporting treatment stratification and precision medicine strategies. **Conclusions**: AI shows promising potential to complement clinical expertise in pediatric IBD by supporting diagnostic assessment, disease monitoring, and therapeutic optimization. However, translation into routine clinical practice remains constrained by methodological heterogeneity, limited pediatric-specific validation, and unresolved ethical and regulatory challenges. Future research should prioritize prospective multicenter pediatric studies, the development of transparent and explainable models, and the integration of AI-based tools into clinically meaningful and patient-centered care pathways.

## 1. Introduction

Inflammatory bowel disease (IBD), encompassing Crohn’s disease (CD), ulcerative colitis (UC), and unclassified IBD (IBD-U), comprises chronic immune-mediated inflammatory disorders of the gastrointestinal tract. Pediatric-onset IBD is frequently characterized by a more extensive and aggressive disease course, with an increased risk of complications and a disproportionate impact on linear growth, pubertal development, and psychosocial well-being [1,2,3]. Compared with adult-onset disease, pediatric IBD differs in terms of disease extent, progression, and monitoring requirements, with children often necessitating repeated objective assessments over prolonged periods. These features increase the relevance of standardized, reproducible, and non-invasive assessment tools, particularly imaging-based and multimodal approaches. The clinical management of pediatric IBD is further challenged by its multifactorial pathogenesis and marked phenotypic heterogeneity, requiring an integrated diagnostic and monitoring strategy that combines clinical, biochemical, endoscopic, histological, and imaging data [3]. In parallel, the adoption of goal-oriented therapeutic paradigms, particularly treat-to-target strategies, has intensified the need for assessment tools that are accurate, reproducible, and capable of capturing disease activity and treatment response longitudinally. In pediatric IBD, the implementation of treat-to-target strategies is often constrained by the limited availability of objective and scalable tools for repeated disease activity assessment, supporting the rationale for exploring data-driven computational approaches [1,2,3].

Within this context, artificial intelligence (AI) has emerged as a promising adjunct in pediatric gastroenterology, particularly in data-intensive domains. Advances in machine learning (ML) and deep learning (DL) have enabled the automated and objective analysis of endoscopic, histopathological, and cross-sectional imaging data, with the potential to reduce observer-dependent variability and improve diagnostic and prognostic precision. Beyond imaging, AI-driven approaches increasingly support therapeutic response prediction, risk stratification, and longitudinal disease monitoring through the integration of clinical variables and patient-reported outcomes [4,5].

Despite this potential, the translation of AI into routine pediatric IBD care remains limited by the scarcity of high-quality pediatric-specific datasets, challenges related to model generalizability and interpretability, and unresolved ethical, legal, and regulatory considerations. Addressing these barriers is essential to ensure that AI technologies are implemented in a safe, equitable, and clinically meaningful manner [6].

This narrative review critically synthesizes current evidence on the application of AI across the diagnosis, monitoring, and management of pediatric IBD, highlighting key advances, limitations, and unmet needs, and identifying priorities to support the responsible integration of AI into pediatric IBD care.

## 2. Materials and Methods

A narrative literature review was conducted using the PubMed (MEDLINE) and Cochrane Library databases, including publications available up to December 2025. The search was guided by key terms related to IBD and AI, with a focus on studies considered clinically relevant to pediatric IBD. The search strategy combined inflammatory bowel disease-related terms with artificial intelligence-related terms, structured to capture applications in diagnosis, disease activity assessment, monitoring, and therapeutic decision-making. Boolean operators were adapted to each database.

Given the limited availability of pediatric-specific evidence, both pediatric and adult studies were included when their methodological approaches, AI architectures, or clinically meaningful endpoints were considered applicable to pediatric IBD. The relevance and limitations of extrapolating adult-derived data to pediatric populations were explicitly considered during synthesis. The study selection and definition of clinically meaningful endpoints were determined a priori by the review team, based on established outcome measures commonly used in pediatric IBD and on their relevance to clinical decision-making.

Eligible publications comprised narrative and systematic reviews, meta-analyses, randomized controlled trials, and prospective or retrospective observational studies addressing AI-based applications in IBD diagnosis, disease activity assessment, monitoring, prognostic stratification, or therapeutic optimization. Case reports, conference abstracts, non-peer-reviewed articles, and studies lacking clear clinical relevance were excluded; only English-language publications were considered.

Titles and abstracts were reviewed for relevance, followed by full-text assessment of selected publications. No formal quality appraisal framework was applied, consistent with the narrative design of this review. A total of 56 articles were included in the final qualitative synthesis. The selected literature was critically appraised with particular emphasis on methodological robustness, dataset characteristics, validation strategies (including external validation when available), model interpretability, and potential applicability to pediatric IBD populations.

## 3. Results

### 3.1. AI in Medicine

AI encompasses computational methods designed to support tasks traditionally requiring human cognitive functions, including pattern recognition, learning, and decision support. In medicine, its growing relevance has been driven by advances in data availability, computational power, and algorithmic development, particularly within machine learning (ML) and deep learning (DL) frameworks [5,7].

A key strength of AI lies in its capacity to identify non-linear patterns and latent relationships within large, heterogeneous datasets, enabling more predictive and personalized approaches to care [7,8]. ML algorithms improve performance through exposure to data, while DL, based on multilayer neural networks, is particularly effective for extracting clinically meaningful features from unstructured data such as medical images and free-text records [5]. Within DL, convolutional neural networks (CNNs) have become the dominant architecture for medical image analysis, demonstrating a robust performance in image classification, segmentation, and disease grading across multiple clinical domains [5].

Importantly, the integration of AI into clinical practice is increasingly framed as a model of augmented intelligence, in which algorithmic systems support rather than replace clinical judgment. This paradigm is particularly relevant in complex, heterogeneous conditions such as IBD, where AI-based tools are best positioned as decision-support systems that enhance efficiency, consistency, and objectivity while preserving clinician oversight and patient-centered care [9].

### 3.2. Applications of AI in the Diagnosis and Management of Pediatric IBD

The diagnosis of IBD relies on a multimodal assessment integrating clinical evaluation with laboratory testing, endoscopy, histology, and cross-sectional imaging [1,2]. Despite this comprehensive approach, diagnostic accuracy remains limited by observer-dependent interpretation, contributing to variability, diagnostic delay, and misclassification, particularly at disease onset. In this context, AI-based approaches, especially those leveraging DL and radiomics, enable rapid, standardized, and reproducible analysis of complex imaging data, with increasing evidence supporting their role in disease detection, phenotyping, and severity assessment [4,9].

Across the following sections, it should be noted that, while pediatric-specific studies are highlighted whenever available, a substantial proportion of AI applications in IBD have been developed and validated in adult or mixed populations.

#### 3.2.1. Endoscopy

Endoscopy remains central to IBD diagnosis and classification but is challenged by interobserver variability, particularly in differentiating UC from CD, and by the time-intensive nature of image interpretation. DL-based systems have demonstrated high diagnostic performance in this setting. Wang et al. developed a CNN-based model (ResNeXt-101) trained on more than 15,000 colonoscopy images, achieving a high accuracy for CD (92.4%), UC (93.4%), and normal mucosa (98.4%) [10]. In a large prospective multicenter study including 1772 participants and over 49,000 images, a CNN-based system outperformed both trainee and expert endoscopists at the patient level, while substantially reducing interpretation time [11]. These findings illustrate the potential of AI-assisted endoscopy to improve diagnostic consistency and workflow efficiency. However, most available evidence derives from adult cohorts, and pediatric-specific validation remains limited due to the paucity of dedicated pediatric studies.

Beyond disease classification, AI has shown robust performance in grading endoscopic disease activity, primarily in adult cohorts. In UC, Stidham et al. trained a deep CNN to distinguish endoscopic remission (Mayo 0–1) from moderate-to-severe disease (Mayo 2–3), achieving an AUC of 0.97 with a strong agreement with expert reviewers (κ = 0.84). Importantly, performance was preserved when applied to full-length colonoscopy videos, supporting the feasibility of AI-assisted activity assessment in real-world clinical practice [12].

Notably, some AI approaches in pediatric IBD integrate endoscopic and histological data, highlighting the potential added value of multimodal analysis for improved diagnostic accuracy (see Section 3.2.3) [13].

#### 3.2.2. Capsule Endoscopy

Video capsule endoscopy (VCE) is particularly valuable for small bowel assessment in suspected or established CD, but its clinical uptake is limited by prolonged reading times and interobserver variability. DL-based systems have demonstrated high diagnostic performance in automated lesion detection within this modality. Klang et al. reported an excellent diagnostic accuracy for the identification of small bowel ulcers and strictures across large VCE datasets, although differentiation between CD-related ulcers and nonsteroidal anti-inflammatory drug-induced lesions remained a relevant limitation [14,15,16].

More recently, the AXARO^®^ DL platform was evaluated in a prospective multicenter cohort of 131 patients with suspected CD. By substantially reducing the proportion of images requiring manual review (to approximately 2.1% of all acquired images), the median reading time decreased to approximately 3 min per examination while preserving a high diagnostic performance [17].

#### 3.2.3. Histology

Histopathological assessment is fundamental to the diagnosis, classification, and prognostic evaluation of IBD, but is inherently limited by interobserver variability, particularly in early disease and borderline phenotypes. AI-based approaches have therefore been explored to standardize histological interpretation, improve reproducibility, and extract quantitative features beyond conventional visual assessment [2,4,9].

In pediatric IBD, Mossotto et al. provided an early proof of concept for AI-driven multimodal classification, demonstrating that the integration of histological and endoscopic data outperformed single-modality approaches in distinguishing UC from CD. In this pediatric cohort (mean age 11.5 years), the combined model achieved higher diagnostic accuracy based on endoscopy or histology alone, underscoring the added value of multimodal integration in pediatric populations [13].

Several DL-based studies conducted predominantly in adult cohorts further support the potential utility of AI-assisted histology. Liu et al. developed and externally validated a DL model to differentiate CD from intestinal tuberculosis using digitized histological slides, achieving good performance in external validation and outperforming non-specialist pathologists [18]. Similarly, Hamamoto et al. demonstrated that a CNN trained on routine histological images could accurately distinguish UC, non-UC proctocolitis, adenocarcinoma, and normal tissue. While these studies are not pediatric-specific, their methodological frameworks are informative for pediatric translation [19].

Beyond diagnostic classification, AI has shown promise in grading histological disease activity and predicting clinical outcomes. Iacucci et al. developed a CNN-based system capable of distinguishing histological remission from active inflammation in UC biopsies using established scoring indices. AI-derived assessments correlated with endoscopic activity and predicted subsequent disease exacerbations, with prognostic performance comparable to expert pathologists [20].

Quantitative, feature-based approaches further extend the scope of AI in histopathology. CNN-driven tissue and cell segmentation has enabled the extraction of interpretable morphometric metrics, such as epithelial cell density, crypt architecture, and immune cell proportions, which correlate strongly with histological severity and accurately identify histological remission. These findings were derived from a mixed adult and pediatric cohort, illustrating how AI can transform complex histological architecture into objective and reproducible biomarkers [21].

Finally, AI-assisted morphometric analysis has demonstrated prognostic potential in CD. CNN-derived histological features obtained at diagnosis have been shown to predict the subsequent development of fibrostenotic and penetrating disease behavior, suggesting a potential role for AI in early risk stratification [22].

These findings suggest that AI-enhanced histopathology may improve diagnostic consistency, standardize disease activity assessment, and provide prognostic insights. Nevertheless, most supporting data originate from adult populations and require dedicated validation in pediatric cohorts, where early and objective phenotyping is essential to support individualized disease management.

#### 3.2.4. Diagnostic Sectional Imaging

Sectional imaging plays a central role in the diagnosis, monitoring, and prognostic assessment of IBD, particularly in CD [1]. However, image interpretation remains partly subjective and affected by interobserver variability. AI-based approaches, including DL and radiomics, have therefore emerged as tools to automate image analysis, extract quantitative biomarkers, and improve diagnostic consistency and prognostic assessment across imaging modalities [4,7,9].

##### Magnetic Resonance Imaging

Magnetic resonance imaging (MRI) has been a major focus of AI applications, particularly for the objective assessment of bowel wall characteristics and intestinal motility. Early studies demonstrated that AI-driven semi-automated segmentation significantly improves the reproducibility of bowel wall thickness measurements compared with manual assessment, with a substantially higher interobserver agreement [23].

More recent work has highlighted intestinal motility as a clinically relevant imaging biomarker. An AI-based analysis of cine-MRI using CNN has been shown to enhance lesion detection compared with static MRI alone and to differentiate patients with CD from controls with moderate accuracy, while identifying additional inflammatory lesions not detected by conventional assessment. Automated motility metrics have also demonstrated stronger associations with clinical symptom burden than subjective radiologist evaluation, supporting their potential clinical relevance [24,25].

Radiomics-based MRI models further extend diagnostic performance. In a pediatric cohort with suspected ileal CD, a ML model based on MRI radiomic features achieved a diagnostic accuracy comparable to experienced radiologists, with additional improvement when combined with clinical data. These findings underscore the potential value of multimodal AI-driven MRI analysis in pediatric populations, particularly for non-invasive disease detection and characterization [26].

##### Intestinal Ultrasound

Intestinal ultrasound is increasingly adopted in pediatric IBD due to its accessibility, safety profile, and suitability for repeated assessments, although operator dependency remains a major limitation. AI-based solutions have shown promise in standardizing ultrasound interpretation and enabling objective disease monitoring. A DL algorithm applied to intestinal ultrasound images from a dataset of 260 pediatric patients, comprising 4565 images (1478 abnormal and 3087 normal), demonstrated a high performance in identifying abnormal bowel wall thickness. In a subset of 612 images, the regions between lumen/mucosa and muscularis/serosa were meticulously annotated, and the AI algorithm was trained to delineate these regions for bowel wall thickness calculation using a 2 mm cutoff. The model achieved a sensitivity of 90.29% and specificity of 93.70%, with a strong agreement compared with specialist measurements. These findings support the potential role of AI-assisted ultrasound as a reliable, non-invasive tool for longitudinal monitoring of disease activity in children, including by less experienced clinicians, in which AI can support image interpretation while image acquisition still relies on the operator [27].

##### Computed Tomography Enterography

Radiomics applied to computed tomography enterography (CTE) has demonstrated particular value in differentiating inflammatory from fibrotic disease, a key clinical challenge in CD management. In a large multicenter study of patients undergoing preoperative CTE and intestinal resection, a radiomic model accurately identified moderate-to-severe intestinal fibrosis and outperformed expert radiologists, with consistent performance across external validation cohorts [28].

Radiomics-based approaches have also been explored for disease phenotyping. ML models derived from CTE features have shown a moderate ability to distinguish CD from UC, with improved performance when radiomic features are combined with clinical variables [29].

Emerging applications include the analysis of visceral adipose tissue using radiomic and three-dimensional DL models. The quantitative assessment of adipose tissue composition has been shown to contribute to disease phenotyping, reflecting the growing recognition of mesenteric fat as a disease-modifying factor in IBD [30].

#### 3.2.5. Multimodal Approach

Across multiple domains, AI models consistently achieve superior performance when quantitative imaging features are integrated with clinical and laboratory data rather than analyzed in isolation. Multimodal approaches, particularly those combining radiomics with biochemical and clinical variables, have consistently shown improved performance in disease characterization, activity assessment, and risk stratification compared with unimodal models [4,5,9].

A representative example is the study by Guez et al., which evaluated a multimodal ML framework for the non-invasive assessment of endoscopic activity in CD using data from the ImageKids multicenter pediatric cohort. Using integrated MRI, biochemical, and clinical data, the optimized fusion model outperformed a guideline-recommended linear MRI-based regression approach in predicting terminal ileal endoscopic activity, with feature importance analysis highlighting disease segment length and inflammatory biomarkers as key contributors [31].

These findings illustrate that AI-based multimodal integration enhances diagnostic and monitoring accuracy by synthesizing complementary sources of information rather than replacing clinical judgment.

### 3.3. Applications of AI in Predicting Therapeutic Response and Treatment Optimization

The heterogeneity of IBD and the variable response to therapy, particularly at disease onset, limit the effectiveness of empirical treatment strategies in pediatric patients. In this context, AI-based models have emerged as valuable tools to support early therapeutic stratification and timely treatment optimization within a precision medicine framework [32,33].

In pediatric UC, AI-driven approaches have shown particular promise. Using histomic features extracted from digital histopathology, ML models have accurately predicted corticosteroid-free remission with mesalazine in treatment-naïve children from the PROTECT2 cohort, enabling the early identification of patients unlikely to respond to monotherapy [32]. Similarly, the integration of mucosal gene expression profiles with clinical variables improved the early prediction of corticosteroid-free remission, outperforming clinical data alone in pediatric cohorts [34].

AI-based prediction models have also been applied to severe UC and biologic therapies. DL frameworks integrating molecular and clinical data have demonstrated the ability to support the individualized selection of rescue therapies, while ML models have shown consistent performance in predicting response to biologic agents across different mechanisms of action [33,35]. Although many of these studies derive from mixed or adult cohorts, their methodological approaches are highly relevant for pediatric translation.

In pediatric CD, AI-based applications extend beyond biologic therapies. The integration of clinical data with microbiome and metagenomic profiles has accurately predicted response to exclusive enteral nutrition, and multi-omic ML approaches linking gene expression patterns to therapeutic outcomes further support the feasibility of AI-driven treatment personalization in pediatric IBD [36,37].

Overall, these findings suggest that AI-based approaches may support a future shift in therapeutic decision-making in pediatric IBD toward more anticipatory and biologically informed strategies. However, most prediction models remain exploratory and require prospective pediatric validation before clinical implementation. Such approaches may enable earlier treatment optimization, improve outcomes, and reduce unnecessary therapeutic exposure in children with IBD.

### 3.4. Patient-Centered Remote Monitoring, Digital Biomarkers, and Telemedicine

The management of IBD is inherently complex due to its relapsing–remitting course and the frequent dissociation between patient-reported symptoms and objective inflammatory activity. In this context, the convergence of AI-driven analytics, digital biomarkers, and telemedicine is reshaping disease monitoring towards a more predictive, proactive, and patient-centered care model [38,39].

Digital biomarkers comprise objectively measured physiological and behavioral data continuously captured through digital devices, including smartphones, wearable sensors, and remote monitoring platforms. Unlike conventional biomarkers, which provide episodic assessments, digital biomarkers offer a longitudinal perspective that may enable the early detection of subclinical changes preceding clinical relapse. Wearable-derived parameters such as heart rate, heart rate variability, physical activity, sleep patterns, and peripheral oxygen saturation have been associated with inflammatory activity in IBD, with prospective data supporting their predictive value when analyzed longitudinally [38]. In a prospective study, an increase of just one beat per minute in daily resting heart rate was associated with a 5% increase in the likelihood of abdominal pain occurring the following day [40]. These findings illustrate the potential of digital biomarkers to provide early, non-invasive indicators of disease activity and to support proactive disease management in pediatric IBD.

The clinical utility of these data has been demonstrated in large prospective cohorts. In the IBD Forecast study, multimodal data derived from wearable devices enabled the prediction of disease flares several weeks in advance, supporting the role of digital biomarkers as early indicators of disease activity, particularly when analyzed using AI-based models capable of handling high-frequency, multidimensional data streams [41].

Mobile health applications further complement remote monitoring by enabling structured symptom tracking, medication adherence support, dietary recording, and the delivery of educational content. In pediatric and adolescent populations, these tools are particularly valuable for fostering self-management skills, promoting responsibility for disease control and treatment adherence, while also supporting the transition to adult care [42]. Daily symptom reporting via mobile applications captures sensitive and frequently underreported information and shows good concordance with clinical assessments, while AI-enhanced platforms can assist in analyzing symptom trajectories and distinguishing inflammatory activity from functional overlap [38].

Digital interventions have also demonstrated benefits in therapeutic adherence. In the SMART IBD^®^ pilot study, AI-supported behavioral prompts and electronic symptom diaries improved short-term medication adherence and patient engagement, with associated improvements in patient-reported outcomes [43].

Telemedicine provides the clinical infrastructure through which these data can be operationalized. Virtual consultations and telemonitoring platforms improve access to care, reduce logistical barriers, and optimize healthcare utilization without compromising clinical outcomes. Platforms such as MyIBDcoach^®^ and TECCU^®^ have been associated with reduced face-to-face consultations and healthcare utilization alongside improved disease control, with remotely collected patient-reported outcomes showing a good agreement with in-person assessments [44,45].

AI plays a central role in integrating these heterogeneous data sources. ML-based approaches enable the fusion of digital biomarkers, patient-reported data, laboratory results, and imaging findings to enhance disease phenotyping, refine risk stratification, and improve the prediction of flares and therapeutic response. More advanced multimodal architectures further illustrate the future direction of remote IBD care, supporting a transition from episodic, reactive management to continuous, data-driven disease monitoring, particularly suited to the long-term needs of pediatric IBD populations [38].

### 3.5. Ethical Challenges and Barriers to AI Implementation

The integration of AI into gastroenterology, particularly in IBD care, offers substantial opportunities to advance precision medicine and improve clinical outcomes. However, its translation into routine practice depends on addressing interconnected ethical, regulatory, and operational challenges that are especially relevant in chronic and heterogeneous diseases such as IBD [6].

A central ethical concern is the risk of perpetuating or amplifying existing health disparities [46]. AI systems are inherently shaped by the data and assumptions underlying their development; without appropriate governance, they may compromise equity, safety, and patient trust. Algorithmic bias can arise from unrepresentative training datasets or flawed design choices. Models that underrepresent specific demographic groups, including pediatric populations, ethnic minorities, or socioeconomically disadvantaged patients, may systematically underperform in these groups. This can lead to inaccurate estimates of disease activity, prognosis, or treatment response and reinforce existing inequities in care [47].

Bias may also be introduced through algorithmic design, even when training datasets appear balanced. The use of inadequate proxy variables is particularly problematic. For instance, employing healthcare expenditure as a surrogate for disease burden has been shown to underestimate medical needs in populations with reduced access to care, despite comparable or greater clinical severity [48]. These observations underscore the need for AI governance frameworks that explicitly address the social and structural determinants embedded in health data and healthcare systems [49].

IBD presents additional disease-specific challenges for AI implementation. Diagnosis, monitoring, and prognostic stratification rely on modalities such as endoscopy, histology, and imaging, which remain partly subjective and affected by interobserver variability. The absence of universally accepted objective reference standards limits model development, validation, and generalizability. This challenge is particularly pronounced in pediatric IBD, where disease phenotype differs from adult forms and high-quality, age-specific datasets remain limited, rendering direct extrapolation from adult-trained models potentially unsafe [50].

Operational barriers further hinder clinical adoption. Health data are often fragmented, heterogeneous, and poorly structured for ML, limiting model robustness and external validation. In addition, AI-based monitoring systems may generate excessive false-positive alerts, contributing to clinician alert fatigue and potentially leading to unnecessary investigations, unwarranted treatment escalation, increased healthcare costs, and avoidable patient anxiety [51].

Finally, the opacity of many proprietary AI systems remains a major obstacle to clinical trust and adoption. These “black box” models provide limited transparency regarding training data, internal architecture, and decision logic, restricting independent evaluations of safety, effectiveness, and fairness [52,53]. In IBD, where therapeutic decisions depend heavily on the interpretation of endoscopic, histological, or imaging findings, limited explainability is particularly problematic and is further compounded by unresolved issues of legal liability in cases of AI-assisted harm.

### 3.6. Future Perspectives and Strategies for Responsible AI Implementation

The future of AI in medicine is increasingly framed as a convergence between human and artificial intelligence, in which AI amplifies clinical expertise rather than replacing medical judgment. This evolution is commonly described as a transition toward AI-augmented healthcare systems, where clinical value emerges from the interaction between algorithmic outputs and clinician interpretation [7,54].

In the short term, AI is expected to primarily enhance efficiency by automating repetitive and high-volume tasks, particularly in data-intensive areas such as endoscopic and imaging interpretation. Over the medium term, advances in data-efficient and multimodal algorithms are anticipated, enabling the integration of imaging, electronic health records, and multi-omic data to support refined disease stratification and precision therapeutic strategies. In the longer term, more adaptive and context-aware systems may facilitate preventive and personalized disease management models. The concept of the digital twin, a dynamic virtual representation of an individual patient used to simulate disease trajectories and therapeutic scenarios, illustrates this potential, although it remains largely investigational [7].

Technological progress must be accompanied by robust governance and regulatory frameworks [46]. Current AI regulation in healthcare remains fragmented, prompting international initiatives such as the FUTURE-AI framework, which defines core principles for trustworthy AI, including fairness, universality, traceability, usability, robustness, and explainability. Effective implementation requires strengthened pre-market evaluation, validation in diverse populations, systematic bias assessment, and post-deployment monitoring, acknowledging the dynamic nature of ML-based systems [55].

Organizational and ethical challenges also persist. Financial and institutional incentives may favor the detection of marginal findings linked to reimbursable interventions, potentially misaligning AI deployment with patient-centered care. Ensuring that AI implementation prioritizes clinical value over economic return is therefore essential. Equally important is investment in workforce training, promoting digital literacy, and enabling clinicians to critically appraise and appropriately integrate AI outputs into clinical decision-making [6,7]. The clinical adoption of AI faces critical legal and financial barriers. Legislatively, systems must ensure compliance with data privacy laws like the General Data Protection Regulation and Health Insurance Portability and Accountability Act, while clear liability frameworks are needed to assign responsibility for AI-driven clinical errors. Financially, the high costs of development and proprietary licensing may restrict these technologies to well-funded centers, potentially worsening socioeconomic disparities in healthcare access [56].

Finally, the development of explainable AI remains central to clinical trust and adoption, particularly in IBD, where therapeutic decisions rely heavily on the interpretation of endoscopic, histological, and imaging findings. Although emerging approaches to improve model transparency are promising, they remain at an early stage and require rigorous validation before widespread clinical adoption [6].

In summary, the responsible integration of AI in IBD will depend on balancing innovation with ethical oversight, positioning AI as a supportive clinical tool that enhances judgment and contributes to sustainable improvements in quality, efficiency, and equity of care.

## 4. Conclusions

Pediatric IBD poses distinctive diagnostic and management challenges due to its clinical heterogeneity, fluctuating disease course, and long-term impact on growth, development, and quality of life. The need to integrate complex and multidimensional data across endoscopy, histology, imaging, and longitudinal monitoring underscores the limitations of conventional assessment strategies and provides a strong rationale for the exploration of advanced computational approaches capable of supporting more objective and consistent clinical evaluation.

The evidence reviewed suggests that AI-based methods have shown promising progress in pediatric IBD, particularly in image-driven applications and multimodal data integration. Across diagnostic and monitoring contexts, ML and DL models have demonstrated the potential to enhance standardization, improve efficiency, and support risk stratification when used as decision-support tools rather than replacements for clinical judgment. Noninvasive techniques and patient-centered digital solutions further extend this potential by enabling continuous monitoring and more individualized disease management, aligning with emerging precision medicine paradigms.

Nevertheless, the translation of AI into routine pediatric IBD care remains limited by methodological, ethical, and organizational challenges. The scarcity of pediatric-specific validation, heterogeneous study designs, and limited model interpretability currently constrain generalizability and clinical trust. Future research should prioritize prospective multicenter pediatric validation, the development of transparent and explainable models, and the assessment of real-world clinical impact through integration into pediatric IBD care pathways. A cautious, evidence-based, and ethically grounded implementation strategy will be essential to ensure that AI evolves as a supportive tool that augments clinical decision-making.

## Data Availability

No new data were created in this narrative review.

## Abbreviations

The following abbreviations are used in this manuscript:

AI	Artificial Intelligence
CD	Crohn’s Disease
CNN	Convolutional Neural Networks
CTE	Computed Tomography Enterography
DL	Deep Learning
IBD	Inflammatory Bowel Disease
ML	Machine Learning
MRI	Magnetic Resonance Imaging
UC	Ulcerative Colitis
VCE	Video Capsule Endoscopy
